# Cascading Failures and Vulnerability Evolution in Bus–Metro Complex Bilayer Networks under Rainstorm Weather Conditions

**DOI:** 10.3390/ijerph16030329

**Published:** 2019-01-24

**Authors:** Fei Ma, Fei Liu, Kum Fai Yuen, Polin Lai, Qipeng Sun, Xiaodan Li

**Affiliations:** 1 School of Economics and Management, Chang’an University, Xi’an 710064, China; mafeixa@chd.edu.cn (F.M.); 2017123045@chd.edu.cn (Q.S.); 2016123083@chd.edu.cn (X.L.); 2 Department of International Logistics, Chung-Ang University, Seoul 06974, Korea; yuenkf@cau.ac.kr (K.F.Y.); polin@cau.ac.kr (P.L.)

**Keywords:** urban public transport, cascading failure, vulnerability, rainstorm, complex network

## Abstract

In recent years, the frequent occurrence of rainstorms has seriously affected urban–public transport systems. In this study, we examined the impact of rainstorms on the vulnerability of urban–public transport systems consisting of both ground bus and metro systems, which was abstracted into an undirected weighted Bus–Metro complex bilayer network (Bus–Metro CBN) and the passenger volume was regarded as its weight. Through the changes in the node scale, network efficiency, and passenger volume in the maximal connected component of the Bus–Metro CBN, we constructed a vulnerability operator to quantitatively calculate the vulnerability of the Bus–Metro CBN. Then, the flow-based couple map lattices (CMLs) model was proposed to simulate cascading failure scenarios of the Bus–Metro CBN under rainstorm conditions, in which the rainstorm is introduced through a perturbation variable. The simulation results show that under the condition of passenger flow overload, the network may have a two-stage cascading failure process. The impact analysis shows that there is a rainstorm intensity threshold that causes the Bus–Metro CBN to collapse. Meanwhile, we obtained the optimal node and edge capacity through capacity analysis. In addition, our analysis implies that the vulnerability of the Bus–Metro CBN network in most scenarios is mainly caused by the degradation of network structure rather than the loss of passenger flow. The network coupling strength analysis results show that the node coupling strength has greater potential to reduce the vulnerability than edge coupling strength. This indicates that traffic managers should prioritize controlling the mutual influence between bus stops (or metro stations) to reduce the vulnerability of the Bus–Metro CBN more effectively.

## 1. Introduction

Public transport is an important part of urban transport and is arguably the basis of a city’s day-to-day operations. However, in recent years, extreme weathers, especially rainstorms, have frequently occurred in many cities, causing detrimental effects on the urban public transport system, resulting in problems such as traffic congestion, traffic accidents, and partial paralysis of transport systems [1]. From the perspective of public health, the impact of rainstorm on the urban public transport system cannot be ignored. On the one hand, when rainstorms occur, urban traffic events are more likely to happen, which will cause residents’ injury or death. On the other hand, the normal running of the vehicles will be disturbed by the impact of rainstorms, for example, the speed of the vehicles may slow down during the rainstorm, and therefore, emit more exhaust gases, which will in turn affects the urban residents’ public health [2,3].

The evolution of urban public transport systems under rainstorm can be described in terms of cascading failures, whereby the failures of certain stops or lines can cause the failures of other stops or lines due to the transfer of passenger flow. To date, some models have been constructed and empirical studies undertaken to analyze the cascading failure processes of different networks, such as the study of cascading failures in India’s power network [4], and the study of the process of cascading failures in a small-world network, scale-free network, weighted complex network and dependent network, etc. [5,6,7,8,9]. Regarding a city’s infrastructure, the cascading failure of urban public transport systems has also been widely researched. For example, Song et al. [10] put forward a method for evaluating the key nodes in an urban public transport network. He et al. [11] proposed an improved cascading failure model of the urban–public transport network based on the capacity-load model and found that cascading failures were more detrimental to the network’s functional integrity rather than to the integrity of its structure.

Vulnerability can cause cascading failures on the urban public transport systems. When the severity of cascading failures increases, the vulnerability of the urban public transport systems also increases. Similarly, the vulnerability of the urban public transport systems decreases as the severity of the cascading failure decreases. To date, there has been no unified definition of transport system vulnerability [12,13,14], with researchers working within two broad categories of this vulnerability. The first category considers that this vulnerability is only related to the failure consequences of certain units (i.e., the components of the system), and is not related to the probability of failure [15,16,17]. For example, Luskova et al. [18] defined vulnerability as a function of exposure, sensitivity, and adaptability to extreme weather, studying the impact of extreme weather on ground transport infrastructure. The second category suggests that vulnerability is related to risk [19,20], subsequently regarding vulnerability as the product of the probability of failure and the consequences of failure. For example, Jenelius and Mattsson [21] proposed a method to analyze road network vulnerability from the latter two angles. This method was able to sort the nodes of a large-scale network according to their importance, and then identify the key lines in different road regions. In line with these studies, we take vulnerability to be an inherent attribute of urban public transport systems, as reflected in the degree of structural and functional changes in an urban public transport system.

In terms of research on vulnerability, numerous scholars have studied the robustness or vulnerability of various real-world systems using the indices of complex networks [22,23,24,25,26], such as computer networks [27], the Internet [28], e-mail networks [29], uncertainty systems [30,31] and transportation network [32], etc. These studies can be divided into two categories according to research methods [33,34]. The first kind of research adopts topology-based method, in which the network load is the topology-based metric such as node degree or node betweenness. Meanwhile, the topology-based studies can be analyzed by analytical method and simulation method. The topology-based analytical method models the transport network without considering node and edge heterogeneities. Some researchers have used this method to analyze the vulnerability of urban public transport systems in L-space and P-space [35,36]. Yin et al. [37] used this method to analyze the structural vulnerability of each segment of Shanghai’s expressway network. The topology-based simulation method models the network with additional consideration of node and edge heterogeneities. Scholars usually use this method to research the performance of different networks under hazards. In these studies, the performance can be measured by the indices of the number of normal and failed components [38,39], the inverse characteristic path length [40], the connectivity loss [41], etc. The second kind of research adopts flow-based method, in which the network load is the particle transported in this network such as current, passengers etc. Soh [42] and Huang [43] applied this method in analyzing the characteristics of Singapore and Beijing transportation networks. Rashidy [44] considered the influence of network structure and passenger flow (the number of passengers) and proposed a new method for assessing the vulnerability of road networks. In general, the flow-based method can capture the flow characteristics of networks and provides more realistic descriptions on the mechanism of network changes.

Although vulnerability studies of the urban public transport systems based on complex network theory have achieved rich results [45,46,47], this traditional research paradigm only includes one type of object and associated relationship, ignoring the heterogeneity (i.e., different types of components and relationships) of complex systems, and arguably leading to the loss of key information pertaining to complex systems. Multi-layer complex networks can contain a variety of objects and relationships, potentially yielding much richer information than traditional single-layer complex networks. Therefore, research focusing on multi-layer complex networks is arguably better suited to reflecting the real-life situation in this context [48,49,50]. Buldyrev et al. [51] found that a large number of nodes need to be removed to cause a complex, single-layer network to collapse but removing only a small number of nodes in a complex, multi-layer network may lead to the collapse of the network structure. Moreover, previous research has also found that the vulnerability of a single-layer network to random node attacks increases with the extensiveness of node degree distribution, while the vulnerability of multi-layer networks shows an opposite relationship. 

Most of the existing studies on the impact of rainstorm on urban public transport system are qualitative, and lack of modeling and quantitative evaluation of the impact of rainstorm. In this study, we focus on the complex bilayer network of bus and metro (Bus–Metro CBN) as the unit of analysis and regard the rainstorm as the external perturbation of the system. Then based on the conventional CMLs model, the flow-based CMLs model is proposed for simulating the cascading failure process in Bus–Metro CBN*s* under rainstorm weather conditions. Finally, we synthesize a vulnerability operator to quantitatively evaluate the impact of rainstorm on the Bus–Metro CBN. Compared with the existing studies, the focus of this study is to establish a dynamic evolution model of the Bus–Metro CBN under rainstorm conditions, and quantitatively evaluate the impact of different intensity rainstorms on the Bus–Metro CBN through the vulnerability indices. In addition, we also explore the impact of node and edge coupling strength on network vulnerability, and the main causes of Bus–Metro CBN vulnerability under different conditions. These findings can provide suggestions for the urban public transportation department for traffic management and emergency repair strategies under rainstorm weather conditions.

The rest of this paper is organized as follows. Section 2 describes the impact of rainstorm on the Bus–Metro CBN. Section 3 presents the cascading failure model of the Bus–Metro CBN operating under rainstorm conditions, together with the method used to measure the vulnerability of this network. Section 4 applies the proposed model and method to an example and analyses the results. Section 5 concludes the entire study.

## 2. The Influence of Rainstorm on Bus–Metro CBN

With global warming, the earth’s surface temperature is rising. Trenberth [52] pointed out that the increase of surface temperature will aggravate surface evaporation. On the one hand, it will lead to drought. On the other hand, the water evaporated into the atmosphere will increase precipitation, which is more prone to rainstorms and floods. Frich et al. [53] found that in the context of climate warming, extreme precipitation events tend to increase in the middle and high latitudes of the Northern Hemisphere. Many studies [54,55,56] have shown that the frequency of extreme precipitation events in the United States, Europe and East Asia has been increasing in the past ~20–30 years. Similar to the global trend, China’s extreme precipitation intensity and extreme precipitation show a growing trend, and rainstorm events have tended to increase. As the intensity of rainstorms vary over time, its impact on the Bus–Metro CBN also varies. According to the research of Papadakis [57], the impacts of rainstorms with different intensities on Bus–Metro CBN*s* are summarized in Table 1.

**Figure 1:**
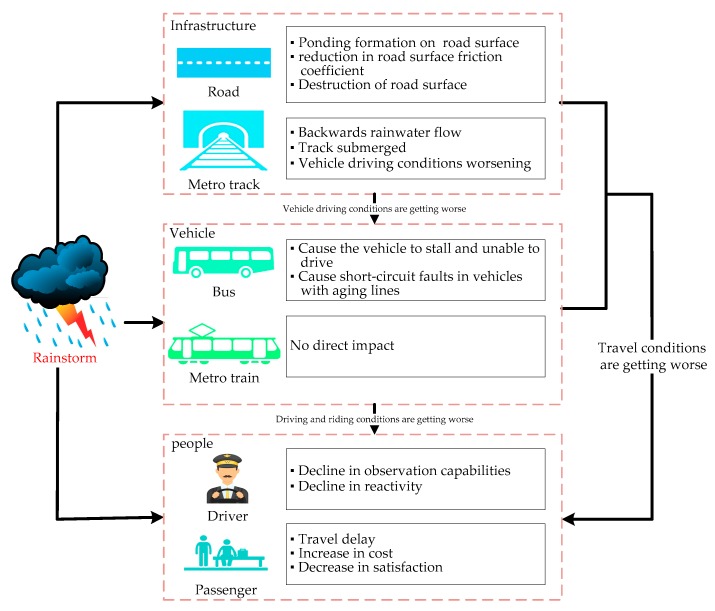
The influence of rainstorms on the Bus–Metro CBN.

The impact of rainstorm on the Bus–Metro CBN can be analyzed from the perspective of roads (or metro train tracks), vehicles and passengers (or drivers), as shown in Figure 1.

### 2.1. The impact of rainstorm on roads and metro train tracks

A rainstorm can cause waterlogging on the road surface, which will reduce the contact between tire and road surface. As a result, it reduces the road surface friction coefficient. Meanwhile, the excessive water accumulation may invade the interior of the asphalt pavement mixed material. Over time, this gradually separates the asphalt membrane from the aggregate surface, which eventually leads to the loss of the bonding force between the asphalt and the aggregate surface, causing the asphalt pavement to wear [58]. Regarding the impact of rainstorms on metro train tracks, a rainstorm could cause rainwater to pour into the metro stations, resulting in stoppage of services in some metro stations [59].

### 2.2. The impact of rainstorm on vehicles

In terms of buses, rainwater can enter the cylinder from the intake and exhaust ports of the engine. Due to the incompressibility of water, the connecting rod of the engine cylinder will be crooked, and the cylinder may be damaged at the same time. The rainstorms may also cause short-circuit faults in vehicles with aging lines [60]. On the other hand, because metro vehicles are mainly operated underground, they are less affected by rainstorms.

### 2.3. The impact of rainstorm on drivers and passengers

From the perspective of drivers, a rainstorm can cause a decline in drivers’ observation abilities, thus significantly increasing the incidence of traffic accidents and decreasing vehicle speed. At the same time, the decline in drivers’ observation abilities can slow down their reaction times, which will further increase the incidence of traffic accidents. From the perspective of passengers, on one hand, rainstorms will increase their difficulty of travel, thereby increasing travel delays and costs. On the other hand, rainstorms will greatly reduce the capacity of Bus–Metro CBN*s*. As a result, the Bus–Metro CBN might not be able to meet all passengers’ travel demands, resulting in queues and congestion [61].

We regard rainstorms as a perturbation to the Bus–Metro CBN, and describe the perturbation with the variable *s* which is calculated as:

(1) $$s=\frac{h}{h_{0}}$$

where *h*_0_ (mm/h) is the intensity threshold of rainstorm, and *h* (mm/h) is the current rainfall intensity, the range of *s* is (0, +∞). From Equation (1), it is obvious that *s* ≥ 1 indicates a rainstorm, 0 < *s* < 1 denote that a normal rainfall and *s* = 0 represents no rainfall. According to the definition of rainstorm by the China Meteorological Administration, the intensity threshold of a rainstorm is 16 mm/h (*h*_0_ = 16 mm/h), or in a cumulative rainfall more than 30 mm for 12 h or 50 mm for 24h (*h*_0_ = 30 mm/12 h or *h*_0_ = 50 mm/24 h).

In this study, we set the state variables *x* and *y* to represent the state of the nodes and edges in the Bus–Metro CBN, respectively. The 0 ≤ *x*, *y* ≤ 1 means that the nodes and edges are in normal operation, and *x* > 1, *y* > 1 indicates that the nodes and edges have failed, respectively. If *x* and *y* follow a uniform distribution in the interval 0 ≤ *x*, *y* ≤ 1, the cumulative distribution functions of *x* and *y* are *F*(*x*) = *x* and *F*(*y*) = *y*. When rainstorms occur, the nodes and edges in the Bus–Metro CBN may fail, which indicates the range of *x* and *y* will be extended to 0 ≤ *x*, *y* ≤ *u*, *u* > 1. Then the cumulative distribution functions of *x* and *y* is $F(x)=\frac{x}{u}$ and $F(y)=\frac{y}{u}$, and the failure probability of nodes and edges can be calculated as follows:

(2) $$\left\{ \begin{array}{l} P(x>1)=1-P(x\leq 1)=1-F(1)=1-\frac{1}{u},\;node\;failure\;probability\\ P(y>1)=1-P(y\leq 1)=1-F(1)=1-\frac{1}{u},\;edge\;failure\;probability \end{array}\right.$$

**Figure 2:**
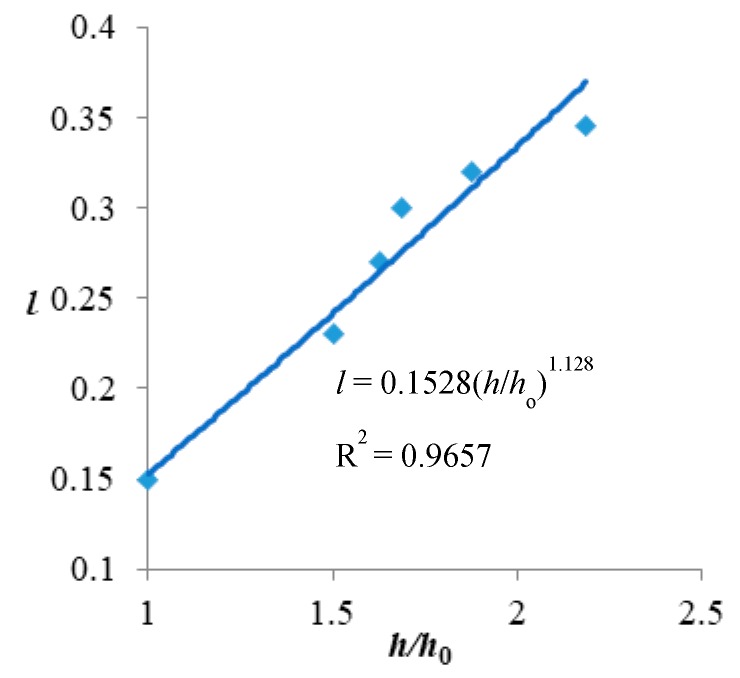
The relationship between traffic flow loss rate *l* and rainstorm intensity *h*, *h*_0_ = 16 mm/h.

According to the research of Dong et al. [62], we obtained the relationship between rainfall intensity *h* (mm/h) and traffic flow loss rate *l* (%). At the same time, Yang’s [63] research on the vulnerability curve of rainstorm and flood disasters shows that the relationship between rainfall intensity and disaster severity exhibits a power law function. Therefore, we use the power law function to fit the obtained relationship between rainstorm intensity and passenger loss rate. The fitting result is:

(3) l=0.1528(hh0)1.128

where *l* is the traffic flow loss rate, *h* is the rainstorm intensity, and *h*_0_ is the intensity threshold of rainstorm. This relationship is shown as Figure 2.

In the Bus–Metro CBN, the traffic flow loss caused by rainstorm can be calculated by multiplying the failure probability of nodes and edges with their traffic volume. In the calculation, the traffic flow loss rate is replaced by the failure probability of nodes and edges. Based on this, we can calculate *u* as follows:

(4) P(x>1)=l⇒1−1u=0.1528(hh0)1.128⇒u=11−0.1528(hh0)1.128=11−0.1528s1.128

Through Formula (4), we can calculate the influence of rainstorm perturbation on node state variable (edge state variable is similar) as follows:

(5) $$\begin{array}{cc} x\to & ux=\frac{x}{1-0.1528s^{1.128}}\\ \mathrm{No}\mathrm{Rainstorm} & \mathrm{Rainstorm} \end{array}$$

where *x* is the node state variable, *s* is the rainstorm perturbation variable, *u* is an intermediate variable.

Many studies [64,65,66] on cascading failure of networks under random or deliberate attacks select one or some nodes and edges randomly or in some way, then disturb them to cause the selected nodes and edges fail to observe the impact of the failed nodes and edges on the whole network. However, it is unreasonable to only consider the failure of some nodes and edges and ignore the impact of rainstorm on other nodes and edges. Therefore, in this study, we believe that rainstorm has an impact on all nodes and edges in the Bus–Metro CBN.

## 3. Models and Methods

### 3.1. Constructing the Bus–Metro CBN

#### 3.1.1. Research Hypothesis

This paper uses the bus and metro subsystems in the Bus–Metro CBN as the research object. In order to simplify the subsystems and facilitate modeling, we propose the following hypothesis about the model construction:

1. Ground buses and the metro are the subsystems of the Bus–Metro CBN, each of which corresponds to one layer of the complex bilayer network.

2. In the ground bus network, a bus stop is considered to be a node, and two nodes are linked by an edge only if there is a direct bus between the corresponding two stops.

3. In the metro network, the metro stations are considered to be the nodes, and two nodes are linked by an edge only if there is a direct train between the corresponding two stations.

4. If the distance between bus stops and metro stations is less than 100 m, these stops, or stations are regarded as transfer stops or transfer stations, i.e., with edges existing between these bus nodes and metro nodes, and these edges represent transfer walking path.

5. If there is more than one bus or metro operating on the route between two bus stops or metro stations, there is seen to be only one edge between the nodes corresponding to the two bus stops or metro stations, in order to avoid duplicate connections.

6. In general, buses or trains can return along the same route they took between one bus stop or metro station to another. Therefore, the direction between nodes is not considered, i.e., the Bus–Metro CBN is constructed to be an undirected network.

7. We do not consider the frequency of buses or trains but only taking into account the connectivity between bus stops or metro stations, as well as the passenger transport capacity at these stops or stations. In other words, the network is a weighted network, and the passenger volume on a particular line is taken as the weight of the corresponding edge.

8. Passengers taking bus or metro will not choose transport modes other than bus and metro. This means if a bus stop (or metro station) failed, all the passengers transported by this bus stop (or metro station) will transfer to the directly linked bus stops (metro stations) and metro station (or bus stop). The transferred passenger volume is determined by the proportion of passengers on the edge to the passengers transported by the node.

#### 3.1.2. Symbolic Description of the Bus–Metro CBN

The Bus–Metro CBN in this study mainly includes conventional ground bus system and Metro system. Due to the heterogeneities of bus stops, bus lines and metro stations, metro lines, i.e., their capacity and load as well as their impacts on the Bus–Metro CBN are different, it is necessary to distinguish the two systems and use bilayer network instead of a single-layer network to represent the composite system of conventional ground bus and metro systems.

1. Complex bilayer network (CBN)

**Figure 3:**
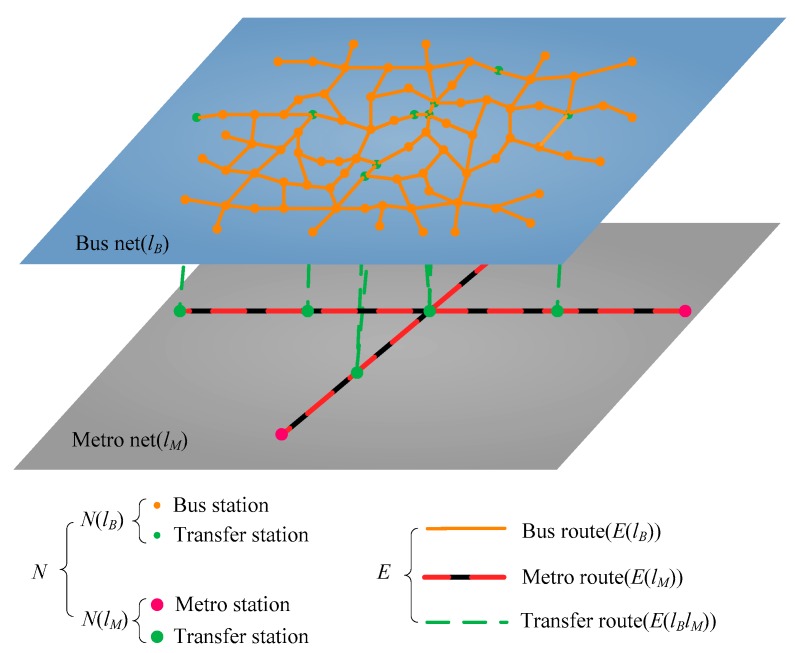
Schematic Bus–Metro CBN model.

The Bus–Metro CBN in this study contains two layers of the ground bus network and the metro network. The corresponding schematic Bus–Metro CBN model is shown in Figure 3. We define the CBN of urban public transport system as follows:

(6) CBN=<N,E,L>

where *N* is a non-empty node set of CBN, and each node represents a bus stop or metro station in the urban public transport system. *E* is the edge set of CBN, including the intra-edges of a single layer network and the inter-edges which link networks of different layers. *L* denotes the layer set of CBN, with each layer representing a single network.

(7) $$N=\{N(l_{B}),N(l_{M})\}$$

where *N*(*l_B_*) and *N*(*l_M_*) indicate the node sets of the ground bus network and metro network, respectively.

(8) $$E=\{E(l_{B}),E(l_{M}),E(l_{B}l_{M})\}$$

where *E*(*l_B_*) represents the intra-edges of the bus network, *E*(*l_M_*) represents the intra-edges of the metro network, and *E*(*l_B_ l_M_*) represents the inter-edges which connect the bus network and metro networks, i.e., *E*(*l_B_ l_M_*) represents the transfer routes in urban public transport system.

(9) $$L=\{l_{B},l_{M}\}$$

where *L* is the set of layers, *l_B_* and *l_M_* refer to the layer of ground bus network and the layer of metro network, respectively.

2. Adjacent matrix of the Bus–Metro CBN

We define *A* as the adjacent matrix of the Bus–Metro CBN, which can be divided into intra-layer adjacent matrices and an inter-layer adjacent matrix. The intra-layer adjacent matrix of *l_B_* is (*l_M_* is similar to *l_B_*):

(10) $$A_{l_{B}}=\left[\begin{array}{cccc} a_{11,l_{B}} & a_{12,l_{B}} & \ldots & a_{1n,l_{B}}\\ a_{21,l_{B}} & a_{22,l_{B}} & \ldots & a_{2n,l_{B}}\\ \ldots & \ldots & \ldots & \ldots \\ a_{n1,l_{B}} & a_{n2,l_{B}} & \ldots & a_{nn,l_{B}} \end{array}\right]$$

where

(11) $$a_{ij,l_{B}}=\left\{ \begin{array}{ll} 1 & n_{i,l_{B}}\;\mathrm{is}\;\mathrm{directly}\;\mathrm{connected}\;\mathrm{with}\;n_{j,l_{B}}\\ 0 & \mathrm{otherwise} \end{array}\right.$$

*n* represents the number of nodes in the ground bus network, *l_B_* is the layer of ground bus network.

The inter-layer adjacent matrix which connects *l_B_* and *l_M_* is:

(12) $$A_{l_{B}l_{M}}=\left[\begin{array}{cccc} a_{11,l_{B}l_{M}} & a_{12,l_{B}l_{M}} & \ldots & a_{1m,l_{B}l_{M}}\\ a_{21,l_{B}l_{M}} & a_{22,l_{B}l_{M}} & \ldots & a_{2m,l_{B}l_{M}}\\ \ldots & \ldots & \ldots & \ldots \\ a_{n1,l_{B}l_{M}} & a_{n2,l_{B}l_{M}} & \ldots & a_{nm,l_{B}l_{M}} \end{array}\right]$$

where

(13) $$a_{ij,l_{B}l_{M}}=\left\{ \begin{array}{ll} 1 & n_{i,l_{B}}\;\mathrm{is}\;\mathrm{directly}\;\mathrm{connected}\;\mathrm{with}\;n_{j,l_{M}}\\ 0 & \mathrm{otherwise} \end{array}\right.$$

*n* and *m* represent the number of nodes in the ground bus network and metro network.

3. Edge weight matrix of Bus–Metro CBN

We define *W* as the edge weight matrix of the Bus–Metro CBN (Formula (13)), where the weight *w_ij_* is the ratio of passenger volume *F_ij_*on edge *e_ij_* to the total passenger volume *F_total_* in the entire network per unit of time.

(14) $$w_{ij}=\frac{F_{ij}}{F_{total}}$$

(15) $$W=\left[\begin{array}{cccc} w_{11} & w_{12} & \ldots & w_{1\left|N\right|}\\ w_{21} & w_{22} & \ldots & w_{2\left|N\right|}\\ \ldots & \ldots & \ldots & \ldots \\ w_{\left|N\right|1} & w_{\left|N\right|2} & \ldots & w_{\left|N\right|\left|N\right|} \end{array}\right]$$

where |*N*| is total number of nodes in the Bus–Metro CBN, |*N*| = *n* + *m*.

4. Passenger flow intensity of nodes and edges in Bus–Metro CBN

The passenger flow intensity can reflect the importance of CBN nodes from a functional perspective. We define *I_i_* as the passenger flow intensity of node *n_i_*, which denotes the number of passengers passing through *n_i_* per unit of time.

(16) $$I_{i}=\sum_{j}F_{ij}$$

where *F_ij_* is the passenger flow volume on edge *e_ij_* per unit of time.

5. Service capability of nodes and edges in Bus–Metro CBN

The service capacity is the maximum number of passengers that can be transported by the Bus–Metro CBN per unit of time. We define *C* as the service capacity matrix of the edges in the Bus–Metro CBN, where *C_ij_* is the maximum number of passengers that can be transported by edge *e_ij_* per unit of time. The service capacity of node *n_i_* is the sum of the service capabilities of the edges directly linked to node *n_i_*, i.e., $C_{i}=\sum_{j}C_{ij}$. According to Motter and Lai’s [67] research on load capacity model (ML model), the capacity of an edge is proportional to its initial load, and the *C_ij_* can be expressed as:

(17) $$C_{ij}=(1+\alpha )L_{ij}$$

where *α* is the tolerance parameter, indicating the additional capacity ratio of the edge *e_ij_*. *L_ij_*is the initial load of edge *e_ij_*, i.e. the initial passenger flow volume on edge *e_ij_*. 

## 3.2. Cascading Failure Model of Bus–Metro CBN under Rainstorm Conditions

### 3.2.1. Passenger Flow Transfer Rules for Cascading Failures in Bus–Metro CBN

**Figure 4:**
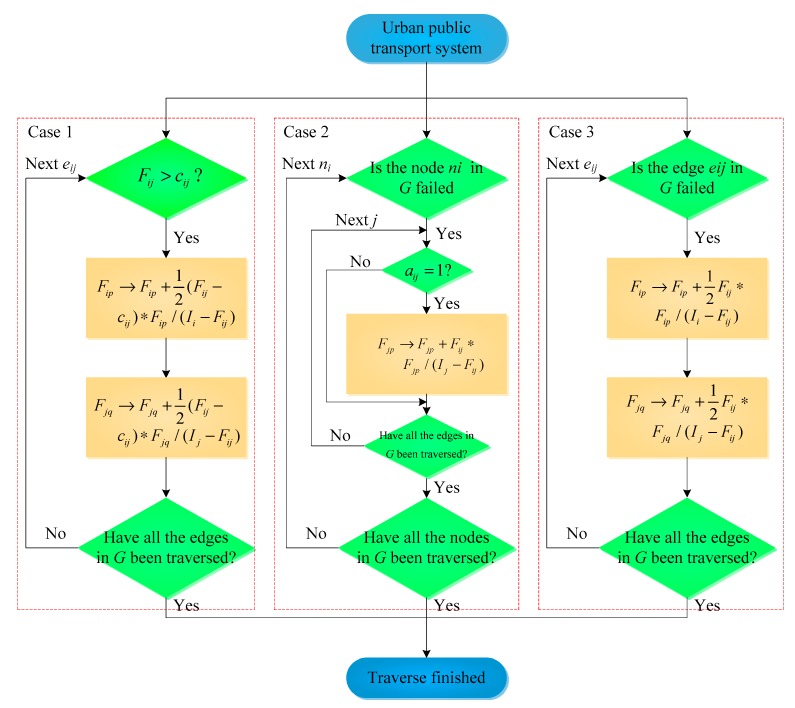
Transfer rules of passenger flow.

The passenger flow transfer rules under cascading failure conditions in the current study could be divided into three categories, i.e., passenger flow overload, node failures and edge failures. We take the ground bus network as an example to introduce these three types of passenger flow transfer rules (Figure 4), and the passenger flow transfer rules in the context of the metro network are similar to that in the ground bus network. These transfer rules are only used for overload or failure of nodes and edges in the largest connected component (*G*) of the Bus–Metro CBN. For other nodes or edges that are isolated due to node or edge failures, we remove these nodes or edges and delete their passengers. The specific transfer steps are as follows:

Case 1. Transfer rules of passenger flow overload at the edges of the ground bus network.

Step 1: Search the overloaded edge *e_ij_* in the largest connected component whose passenger flow *F_ij_* exceeds its service capacity *C_ij_*.

Step 2: Remove the edge *e_ij_*.

Step 3: The passenger flow on *e_ij_* will transfer to the edges that directly link to node *n_i_* and node *n_j_* (the flow will transfer to these edges only if the state of these edges is normal). Because in the real Bus–Metro CBN, passengers would be much more likely to choose the nearby transit services to escape from the confusion and accident as soon as possible when a bus stop (or metro station) or line suffers from disaster or attack.

Step 4: The volume of passenger flow at the edge *e_ip_* that directly link to node *n_i_* will change as follows: *F_ip_*→*F_ip_* + ∆*F_ip_*, where ∆*F_ip_ = F_ij_* · *F_ip_*/(*I_i_ − F_ij_*), where the change of passenger volume at node *n_j_* is similar to that at node *n_i_*.

Step 5: Search other overloaded edges and repeat steps 1 to 4 until all the edges are traversed.

Case 2. Passenger flow transfer rules in the case of node failures in the ground bus network.

Step 1: Search for the failed node *n_i_* in the largest connected component which is a node cannot be operated normally, i.e., passengers cannot take bus or metro at this node, and bus and metro cannot pass this node).

Step 2: Remove node *n_i_* and the edges link to *n_i_*.

Step 3: The flow *F_ij_* will transfer to the nodes *n_j_* which directly link to node *n_i_* (this will occur only if the states of these nodes are normal).

Step 4: The volume of passenger flow at edge *e_jp_* that directly link to node *n_j_* will change as follows: *F_jp_*→*F_jp_*+ ∆*F_jp_*, where ∆*F_jp_ = F_ij_* · *F_jp_*/(*I_j_ − F_ij_*).

Step 5: Search for other failed nodes and repeat steps 1 to 4 until all the nodes are traversed.

Case 3. Passenger flow transfer rules in the case of edge failures in the ground bus network.

Step 1: Search for the failed edge *e_ij_* in the largest connected component which is an edge cannot be operated normally, i.e. bus and metro cannot drive on this edge, and passengers cannot walk on this edge).

Step 2: Remove edge *e_ij_*.

Step 3: The passenger flow on edge *e_ij_* will transfer to the edges that directly link to node *n_i_* and node *n_j_* (only if the states of these edges are normal).

Step 4: The passenger volume of edge *e_ip_* will change as follows: *F_ip_*→*F_ip_* + ∆*F_ip_*, where ∆*F_ip_ = F_ij_* · *F_ip_*/(*I_i_ − F_ij_*), and the changes of passenger volume on the edges directly link to node *n_j_* are similar to that on the edges directly link to node *n_i_*.

Step 5: Search for other failed edges and repeat steps 1 to 4 until all the edges are traversed.

### 3.2.2. Cascading Failure Model based on CMLs

The cascading failure of Bus–Metro CBN can be explained by self-organized criticality theory. This theory regards that the system which is composed of a large number of interacting components will naturally develop towards a self-organized critical state. When the system is in this state, even a small disturbance event may cause a series of catastrophes [68]. Generally, it is considered that power-law distribution is the sign of self-organized critical system, and the self-organized critical phenomenon is the dynamic reason for the formation of the power-law distribution [69,70]. Many empirical studies find that the node degree distribution of transport network has power-law characteristics, such as the Beijing transit network [71], the public transport networks in Poland [72], the 33 metro systems in different cities [73], etc. Based on these studies, the Bus–Metro CBN can be regarded as a self-organized system and small local changes can be magnified and extended to the entire network, which may cause avalanches in the system [74]. To explain this self-organized critical phenomenon, many scholars have proposed different cascading failure models according to different research objects, such as Load (or Flow) Capacity model [75], Binary Impact model [76], Sand model [77], Couple Map Lattices model [78], etc.

Couple Map Lattices (CMLs) is a dynamic model that describes the complex spatio-temporal behavior of non-linear systems [79]. Because of the excellent spatio-temporal chaos characteristics and easy to analyze and numerically process, this model has been widely applied to population studies [80], liquid flow studies [81], chemical reactions [82] and biological networks [83], etc. In recent years, some researchers have applied the CMLs model to the study of cascading failure in complex networks [84,85]. In this study, we use the CMLs model to simulate the change of Bus–Metro CBN under rainstorm conditions. The research object of this study is the weighted Bus–Metro CBN, where the weight is represented by the volume of passenger flow at the edges. Therefore, the cascading failure of this Bus–Metro CBN is based on the topological structure of the network and the distribution of passenger flow, which could be expressed using the coupling function. Following this, and based on the CMLs model presented by Huang, Peng and Ren [64,65,66], we proposed a flow-based cascading failure model of the Bus–Metro CBN under rainstorm conditions. The model includes two parts of the node failure model and the edge failure model, which are as follows:

1. Cascading failure model of nodes in Bus–Metro CBN

(18) $$x_{i}(t+1)=\left|(1-\varepsilon_{1})f\left(x_{i}(t)\right)+\varepsilon_{1}\sum_{j=1,j\neq i}^{{\left|N\right|}_{G}}\frac{F_{ij}f\left(x_{j}(t)\right)}{I_{i}}\right|,=1,2,\ldots ,\left|N\right|$$

where *t* and (*t* + 1) denote the time step. *x_i_*(*t* + 1) represents the state variable of node *n_i_* at the (*t* + 1) time step, which can be used to determine whether the corresponding bus stops (or metro stations) are able to provide a travel service. We used an absolute value notation in Equation (16) to guarantee the non-negative state of each node. We then assumed that 0 ≤ *x_i_*(*t* + 1) ≤ 1 represents the normal state of node *n_i_* at the (*t* + 1) time step. Meanwhile, *x_i_*(*t* + 1) > 1 represents that node *n_i_* failed. The variable *ε*_1_ represents the node coupling strength of the Bus–Metro CBN, if *ε*_1_ = 0.2, there will be 20% state changes of a node come from the impact of their neighbor nodes’ states. In real life, *ε*_1_ indicates that the influence of the operation of a bus stop (or metro station) on the operation of its neighbor bus stops (or metro stations). |*N*| is the number of nodes in the Bus–Metro CBN. *F_ij_* and *I_i_* represent the passenger flow volume of edge *e_ij_* and node *n_i_*, respectively. In line with previous research [84,85,86,87], the traffic flow has chaotic characteristics and can be simulated with the chaotic logistic map. Therefore, in this study *f* was chosen to be the chaotic logistic map: *f*(*x*) = 4*x*(1 − *x*), when 0 ≤ *x* ≤ 1, 0 ≤ *f*(*x*) ≤ 1. The function *f* indicates that a single node in the Bus–Metro CBN is a chaotic dynamic system, which can simulate the local dynamic changes of node capacity [88].

Rainstorm will affect the normal state of all nodes in the Bus–Metro CBN. According to the analysis in Section 2.1, we set *s* (the intensity of rainstorm) as the external disturbance, *s* ≥ 1, and assumed that rainstorm will affect all the nodes. Then the state variable of node *n_i_* can be expressed as follows:

(19) $$x_{i}(t)=\frac{1}{1-0.1528s^{1.128}}\left|(1-\varepsilon_{1})f\left(x_{i}(t-1)\right)+\varepsilon_{1}\sum_{j=1,j\neq i}^{{\left|N\right|}_{G}}\frac{F_{ij}f\left(x_{j}(t-1)\right)}{I_{i}}\right|$$

Equation (17) is used to simulate the impact of rainstorm on the nodes in Bus–Metro CBN. The *x_i_*(*t* − 1) is multiplied by $\frac{1}{1-0.1528s^{1.128}}$ to simulate that rainstorm will increase the probability of node failure, rather than necessarily cause the node to fail. In real world, the rainstorm usually only causes some bus stops (or metro stations) to fail and not all nodes to fail. Moreover, with an increase in *s*, node *n_i_* will be more likely to induce *x_i_*(*t*) > 1, which will cause more bus stops (or metro stations) to fail. When node *n_i_* fails, node *n_i_* as well as its adjacent edges will be removed, following which the state variable will remain +∞. The passengers passing through node *n_i_* will transfer to the adjacent bus nodes and metro nodes, as per the transfer rule shown in Figure 4.

We can use Equations (16) and (17) to mimic the cascading failure of nodes in Bus–Metro CBN under rainstorm. At first, all the nodes are normal, their state values are in (0,1), and their state changes can be described by Equation (16). Then at the beginning of simulation, a rainstorm happened (regarded as perturbation which will affect every node), which cause some nodes failed according to Equation (17). Next, the state changes will propagate to the nodes which link to the failed nodes according to Equation (16), which can lead to a new round of node failure. This process is repeated and may eventually cause the collapse of the entire network. 

2. Cascading failure model of edges in Bus–Metro CBN

Nodes are the basic elements of the network and are undoubtedly extremely important to the network. Many scholars have researched the cascading node failures in complex networks [89,90,91,92,93,94]. Similar to nodes, edges are also the basic elements of the network. In the real world, many infrastructure systems have some important routes, such as main transmission line in power-grid system, main lines in Internet systems and main road in transportation systems [95,96]. These routes usually have large flows of volume and connect different large components in these systems. Once these routes fail, the entire system may split into isolated parts, some pairs of nodes may become unreachable and the connectivity of the system will be greatly reduced. Therefore, it is also important to consider the edge failures in the network. In Bus–Metro CBN, roads may become impassable due to waterlogging, in turn leading to ground bus diversions or bus outage, and eventually potentially causing cascading failures in the Bus–Metro CBN. In addition, when the node fails, its connected edges will also fail. As long as there is a failure of a node connected to the edge, the edge will also fail. Therefore, the state value of the edge depends not only on the edge itself, but also on the two nodes connected by this edge. Therefore, we can use the maximum value of the two nodes’ state values and the edge’s calculated state value as the state value of the edge, the cascading failure model relating to edges can be expressed as follows:

(20) $$y_{ij}(t+1)=\max\left\{\left|(1-\varepsilon_{2})f\left(y_{ij}(t)\right)+\frac{\varepsilon_{2}}{2}\left(\sum_{p=1,p\neq i,j}^{{\left|N\right|}_{G}}\frac{F_{ip}f\left(y_{ip}(t)\right)}{I_{i}}+\sum_{q=1,p\neq i,j}^{{\left|N\right|}_{G}}\frac{F_{jq}f\left(y_{jq}(t)\right)}{I_{j}}\right)\right|,x_{i}(t),x_{j}(t)\right\}$$

where *t* and (*t* + 1) denote the time step, *x_i_*(*t*) and *x_j_*(*t*) are the state value of node *n_i_* and *n_j_* at *t* time step, which means if the node *n_i_* or *n_j_* that directly linked to *e_ij_* failed, the edge *e_ij_* will also fail. *y_ij_*(*t* + 1) denotes the state variable of edge *e_ij_* at (*t* + 1) time step, which indicates whether the bus line (or metro line) can transport passengers normally. Then, we use 0 ≤ *y*_ij_(*t* + 1) ≤ 1 to represent the state of edge *e_ij_* is normal, and *y*_ij_(*t* + 1) > 1 to represent edge *e_ij_* has failed. *ε*_2_ represents the edge coupling strength, which indicates how much state changes of an edge come from its neighbor edges. In the real world, *ε*_2_ is the impact degree of a bus line (or metro line) on its neighbor bus lines (or metro lines). |*N*| is the number of nodes in a Bus–Metro CBN. *F_ip_*, *F_jq_*, *I_i_* and *I_j_* represent the passenger flow volume of edges *e_ip_* and *e_jq_*, and nodes *n_i_*, *n_j_*, respectively. Equation (18) indicates that the state of edge *e_ij_* is affected by its own disturbance and by the state of nodes *n_i_* and *n_j_*. In other words, as long as one of the two nodes fails, edge *e_ij_* will also fail. At the same time, if the edge *e_ij_* is normal at *t* time step, then the nodes *i* and *j* must be normal at *t* time step, which means Equation (18) must contain three equations if the edge *e_ij_* is normal at *t* time step. Subsequently we can calculate the state value of *e_ij_* at *t* + 1 time step. Similar to what happens with the nodes, we set a rainstorm *s* as an external disturbance (s ≥ 1) which also affects the normal state of all edges. The state variable of edge *e_ij_* at the *t* time step can be expressed as follows:

(21) $$y_{ij}(t)=\max\left\{\begin{array}{l} \frac{1}{1-0.1528s^{1.128}}\left|(1-\varepsilon_{2})f\left(y_{ij}(t-1)\right)+\frac{\varepsilon_{2}}{2}\left(\sum_{p=1,p\neq i,j}^{{\left|N\right|}_{G}}\frac{F_{ip}f\left(y_{ip}(t-1)\right)}{I_{i}}+\sum_{q=1,p\neq i,j}^{{\left|N\right|}_{G}}\frac{F_{jq}f\left(y_{jq}(t-1)\right)}{I_{j}}\right)\right|,\\ \frac{1}{1-0.1528s^{1.128}}x_{i}(t-1),\frac{1}{1-0.1528s^{1.128}}x_{j}(t-1) \end{array}\right\}$$

Equation (19) was used to simulate the influence of a rainstorm on the edges. Due to the fact when a rainstorm occurs, all the nodes and edges will be affected, the *x_i_*(*t* − 1) and *x_j_*(*t* − 1) should also be multiplied by $\frac{1}{1-0.1528s^{1.128}}$. At *t* time step, the state variable of edge *e_ij_* depends on two factors. The first is the state of the adjacent edges of *e_ip_* and *e_jq_*, nodes *n_i_* and *n_j_* at the (*t* − 1) time step. The second factor is the intensity *s* of a rainstorm. If *y_ij_* > 1 at (*t* − 1) time step, then *y_ij_*(*t*) > 1, and the state variable of edge *e_ij_* will remain greater than 1 thereafter. If 0 ≤ *y_ij_*≤ 1 at (*t* − 1) time step, then the state variable of edge *e_ij_* at *t* time step can be calculated using Equation (19). Equation (19) shows that the probability of failure of edges increases with an increase in the severity of a rainstorm, which is consistent with the actual situation. When edge *e_ij_* fails, *e_ij_* will be removed from the network. The passengers transported by edge *e_ij_* will thus transfer to the adjacent nodes and edges, as per the transfer rule shown in Figure 4.

Equations (18) and (19) can simulate the edge cascading failures in Bus–Metro CBN under a rainstorm. In the beginning, all edges are normal, and their state values are in (0,1). Then rainstorms occur, according to the calculation by Equation (19), some edges may fail and other edges will still work normally. Next, the state values of all normal edges will be recalculated by Equation (18), and the failed edges’ state will propagate to their neighbor edges. This process will continue until the entire network is re-stabilized or eventually collapses.

The failures in Bus–Metro CBN*s* may be caused by natural hazards such as rainstorms, or it may be caused by deliberate attacks such as terrorist attacks. The impact of natural hazards on the Bus–Metro CBN can be regarded as a random attack, while the deliberate attacks such as terrorist attacks are targeted attacks. In this study, the proposed flow-based CMLs model is only applicable to the simulation of natural hazards to random attacks on the Bus–Metro CBN. The bus stop (line) and metro station (line) are heterogeneous due to the capacity and passenger intensity of bus stop (line) and metro station (line) are different. Then according to the proposed CMLs model, the bus stop (lines) and metro station (line) will show different failure behaviors in the simulation. So, we only distinguish the bus traffic and metro traffic in the node (edge) properties rather than in the CMLs model.

## 3.3. Vulnerability Analysis of Bus–Metro CBN under Rainstorm

Previous research on the vulnerability of complex networks is mainly based on the topology changes. The vulnerability of networks is reflected by the sensitivity of the node scale in a maximum connected subgraph and the efficiency of the entire network to different attack strategies [40,41,88,89]. In this study, we explore the vulnerability of Bus–Metro CBN under rainstorms, not only considering the changes in the network topology (i.e., the number of nodes in the network and the connection relationships between network nodes) under rainstorm conditions, but also considering the changes in network functions (i.e., the function of transporting passengers). Therefore, on the basis of the preceding two indices of topology changes in the networks, the indicator of passenger flow volume of the entire network is increased to reflect the functional changes of Bus–Metro CBN under a rainstorm.

1. Node scale changes in a maximal connected subgraph of the Bus–Metro CBN

The maximal connected subgraph of the Bus–Metro CBN represents the component which contains the largest number of nodes in all components of Bus–Metro CBN. We used |*N*|_0_ to denote the node scale of the maximum connected subgraph of the initial Bus–Metro CBN, and |*N*|*_T_* to represent the node scale of the maximum connected subgraph when Bus–Metro CBN is finally stable. Thus, the node scale changes in the maximal connected subgraph of the Bus–Metro CBN can be described as:

(22) $$\eta (\left|N\right|)=\frac{{\left|N\right|}_{0}-{\left|N\right|}_{T}}{{\left|N\right|}_{0}}$$

where *η*(|*N*|) is the node scale change in the maximal connected subgraph of the Bus–Metro CBN, *η*(|*N*|)∈[0,1].

2. Whole-network efficiency changes of the Bus–Metro CBN

Efficiency can reflect the tightness of node connection in a network. The efficiency *R_ij_* of node *n_i_* and node *n_j_* in the Bus–Metro CBN is defined as the reciprocal of the shortest path (the path that contains the minimum number of edges in all paths which link node *n_i_* to node *n_j_*), i.e., *R_ij_*= 1/*d_ij_*. The efficiency *R* of the CBN is taken as the average value of each node’s efficiency:

(23) $$R=\frac{1}{\left|N\right|(\left|N\right|-1)}\sum_{i\neq j}\frac{1}{d_{ij}}$$

where |*N*| represents the number of nodes in the Bus–Metro CBN, and *d_ij_* denotes the number of edges on the shortest path between nodes *n_i_* and *n_j_*. When nodes *n_i_* and *n_j_* are not connected, *d_ij_* = ∞. The |*N*|(|*N*| − 1) represents the no duplication of node logarithm.

According to Equation (21), the whole-network efficiency changes of the Bus–Metro CBN can be calculated as:

(24) $$\eta (R)=\frac{R_{0}-R_{T}}{R_{0}}$$

where *R*_0_ denotes the whole-network efficiency of the initial Bus–Metro CBN, and *R_T_* represents the whole-network efficiency when the Bus–Metro CBN is finally stable, 0 ≤ *η*(*R*) ≤ 1.

3. Changes in the total volume of passenger flow in the largest connected component of Bus–Metro CBN

The total volume of passenger flow in the Bus–Metro CBN is the sum of passengers on all edges in the largest connected component (*G*) of Bus–Metro CBN. The total passenger flow volume *F_total_* can thus be calculated as:

(25) $$F_{total}=\sum_{i\in G}^{{\left|N\right|}_{G}}\sum_{j\in G,j>i}^{{\left|N\right|}_{G}}F_{ij}$$

where |*N*|*_G_* is the number of nodes in the largest connected component of Bus–Metro CBN, and *F_ij_* is the passenger flow volume on edge *e_ij_*.

According to Equation (23), we can calculate the changes in the total volume of passenger flow *η*(*F*) in the Bus–Metro CBN as:

(26) $$\eta (F)=\frac{F_{0}-F_{T}}{F_{0}}$$

where *F*_0_ denotes the total volume of passenger flow in the initial Bus–Metro CBN, and *F_T_* represents the total volume of passenger flow in the largest connected component when the Bus–Metro CBN is finally stable, *η*(*F*)∈(0,1).

4. Vulnerability operator construction of Bus–Metro CBN

The vulnerability of Bus–Metro CBN in this study was measured in terms of node scale changes in the maximum connected subgraph, efficiency changes in the whole network, and changes in the total volume of passenger flow. Therefore, the vulnerability operator *V* can be expressed as:

(27) $$V=\lambda_{1}\times \eta (\left|N\right|)+\lambda_{2}\times \eta (R)+\lambda_{3}\times \eta (F)$$

where *η*(|*N*|), *η*(*R*), and *η*(*F*) denote node scale changes in the maximum connected subgraph, efficiency changes in the whole network, and changes in the total volume of passenger flow, respectively. *λ*_1_, *λ*_2_ and *λ*_3_ represent the weight of *η*(|*N*|), *η*(*R*) and *η*(*F*), respectively. These weights indicate the importance of topological structure and function in the Bus–Metro CBN. *λ*_1_, *λ*_2_, *λ*_3_∈(0,1), and *λ*_1_ + *λ*_2_ + *λ*_3_ = 1. *V* is the vulnerability of the Bus–Metro CBN, *V*∈[0,1]. The larger the value of *V*, the more vulnerable the Bus–Metro CBN, i.e., the topological structure and function of the Bus–Metro CBN change sharply. 

## 4. Case Study

### 4.1. Research Area

**Figure 5:**
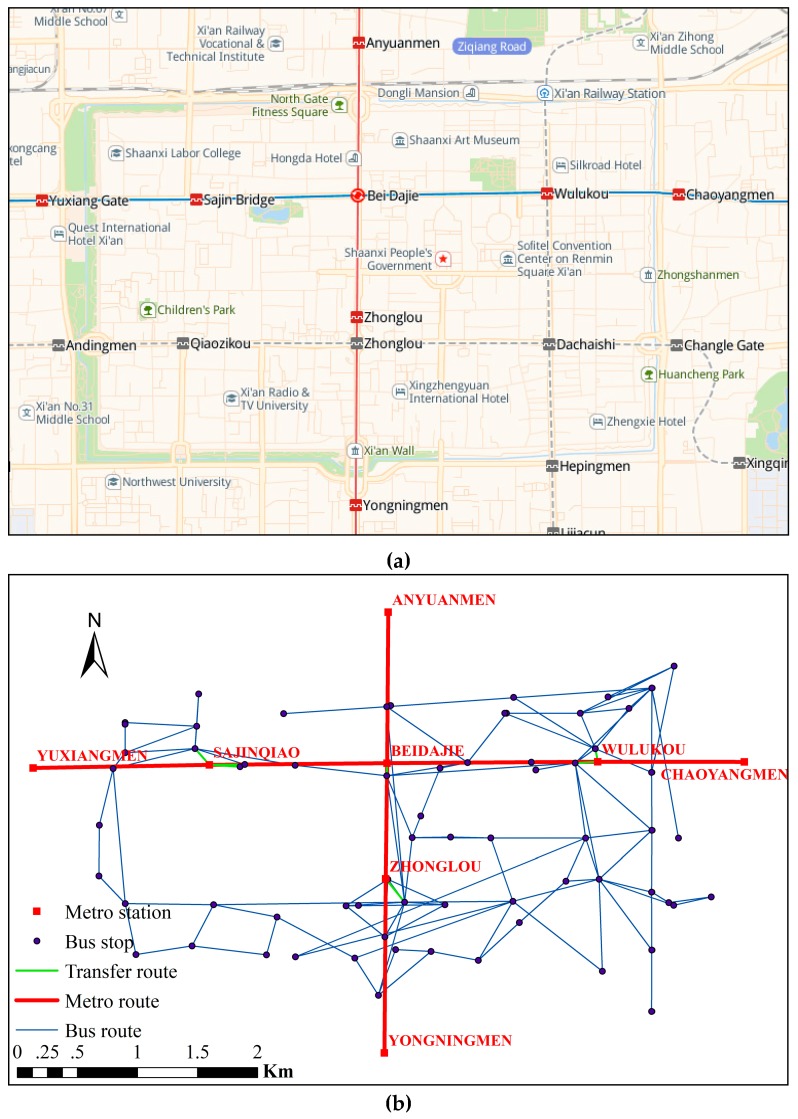
Bus–Metro CBN of the area encircled by Xi’an City wall. (**a**) Actual Bus–Metro CBN traffic network. (**b**) Corresponding Bus–Metro CBN topological network.

**Figure 6:**
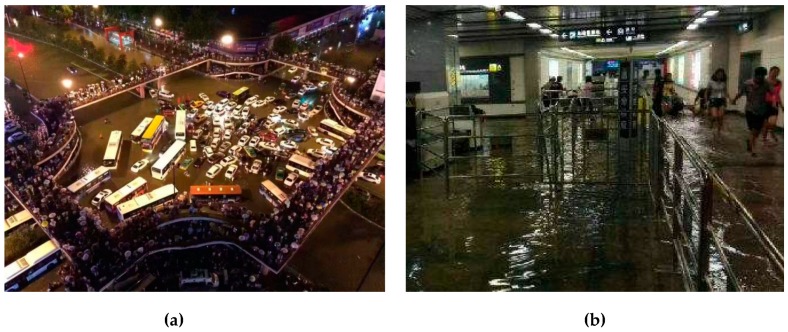
Bus–Metro CBN of Xi’an City after a rainstorm on 24 July 2016. (**a**) Bus stop after a rainstorm. (**b**) XIAOZHAI metro station after a rainstorm.

The research area selected for this study is that encircled by Xi’an City wall (the models and research methods in this study can also be applied to the Bus–Metro CBN in other cities with buses and metros), located in Shaanxi, China (Figure 5a); i.e., the geographical center of Xi’an City. The transportation network there is relatively congested. The Bus–Metro CBN encircled by the Xi’an City wall mainly consists of the ground bus and metro, which contains 54 bus stops, 8 metro stations, 93 bus lines operating between bus stops, and 2 metro lines running between the metro stations. Key data pertaining to the Bus–Metro CBN in this area is shown in Table 2 (the data acquisition from Baidu map 2017). Normally, this Bus–Metro CBN operates in a stable manner, but when rainstorms occur, the Bus–Metro CBN becomes relatively vulnerable. For instance, a rainstorm on 24 July 2016 caused the efficiency of the ground bus network in this area to decline sharply, and the rainwater pouring into the XIAOZHAI metro station resulted in the closure of this metro station. This then caused the public transport in this area to almost collapse (Figure 6). Therefore, choosing the area encircled by Xi’an City wall as the research area, within which to explore the vulnerability of Bus–Metro CBN under rainstorm conditions, enabled us to find ways of optimizing this Bus–Metro CBN and preventing, as well as controlling its vulnerability. 

The Bus–Metro CBN of the research area is mapped out in Figure 5a. The corresponding Bus–Metro CBN was constructed according to the method proposed in Section 2.1 and is shown in Figure 5b. Then, using UCINET 6.0, we obtained the node degree, shortest path and average path length, clustering coefficient, and average clustering coefficient of the Bus–Metro CBN. The results are shown in Table 3. 

As shown in Table 3, the Bus–Metro CBN constructed in this study contains inter-layer edges, i.e., edges that connect the bus network and the metro network, resulting in the average degree of this Bus–Metro CBN being higher than that of the bus network and metro network. The average path length of the Bus–Metro CBN was found to be greater than that of the metro network and smaller than that of the bus network. This is mainly due to the small number of nodes in the metro network. The clustering coefficient (which indicates the degree of node aggregation) of the Bus–Metro CBN emerged as smaller than that of the bus network, indicating that the local clustering degree of this Bus–Metro CBN has declined. The clustering coefficient of the metro network was 0, indicating that there were no edges between any two adjacent nodes of each node in this network. 

### 4.2. Scenario Descriptions

According to data of Xi’an Public Transport Corporation in 2017, the daily average passenger volume of ground buses in the area encircled by Xi’an City wall was about 400,000 people, and the daily average passenger volume of the metro was about 150,000 people. We allocate passengers according to the number of bus lines on the edges (the number of bus lines passing through this edge). Specifically, the passenger volume on an edge is obtained by multiplying the ratio of the number of bus (or metro) lines on the edge to the number of bus (or metro) lines by the total passenger volume. Then the passenger volume of nodes can be calculated by Equation (14). In addition, we set the initial state of each node or edge as the random value of the interval (0,1), and set up four types of simulation scenarios containing the impacts of rainstorm intensity, capacity tolerance, node coupling strength, and edge coupling strength on the Bus–Metro CBN. We then used the approach of variable-controlling to explore these impacts.

In the first type of simulation scenario, we set the *ε*_1_, *ε*_2_ and *α* as constants, and the *s* as a perturbation variable at the beginning of simulations to study the impact of rainstorm intensity on the Bus–Metro CBN under rainstorm conditions. For example, according to the data of Xi’an Meteorological Bureau, the rainfall in Xi’an on 24 July 2016 was 110 mm. According to Equation (1), this rainstorm can be transformed into the perturbation variable where *s* = 2.2. Then the perturbation *s* is substituted into Equations (17) and (19) to calculate the state changes of nodes and edges. Based on this, we can analyze the impact of rainstorm on the Bus–Metro CBN using Equations (20)–(25). In the second simulation scenario, we set the *ε*_1_, *ε*_2_ and *s* as constants, and *α* as a variable to observe the changes of cascading failure process with the capacity tolerance. In the third type of simulation scenario, we set the *s*, *α* and *ε*_2_ as constants, and the *ε*_1_ as a variable to examine the impact of node coupling strength on the Bus–Metro CBN under rainstorm conditions. In the fourth type of simulation scenario, we set the *s*, *α* and *ε*_1_ as constants, and the *ε*_2_ as a variable to observe the impact of edge coupling strength on the cascading failures in Bus–Metro CBN.

The specific variable values in these scenarios are shown in Table 4. In line with the various simulation scenarios and the flow-based CML model established in Section 3, we simulated the evolutions of topological structure and passenger flow in this Bus–Metro CBN under rainstorm conditions. Then we recorded the changes of the Bus–Metro CBN and present the results in Section 4.3.

### 4.3. Vulnerability Analysis of Bus–Metro CBN under Rainstorm Conditions

**Figure 7:**
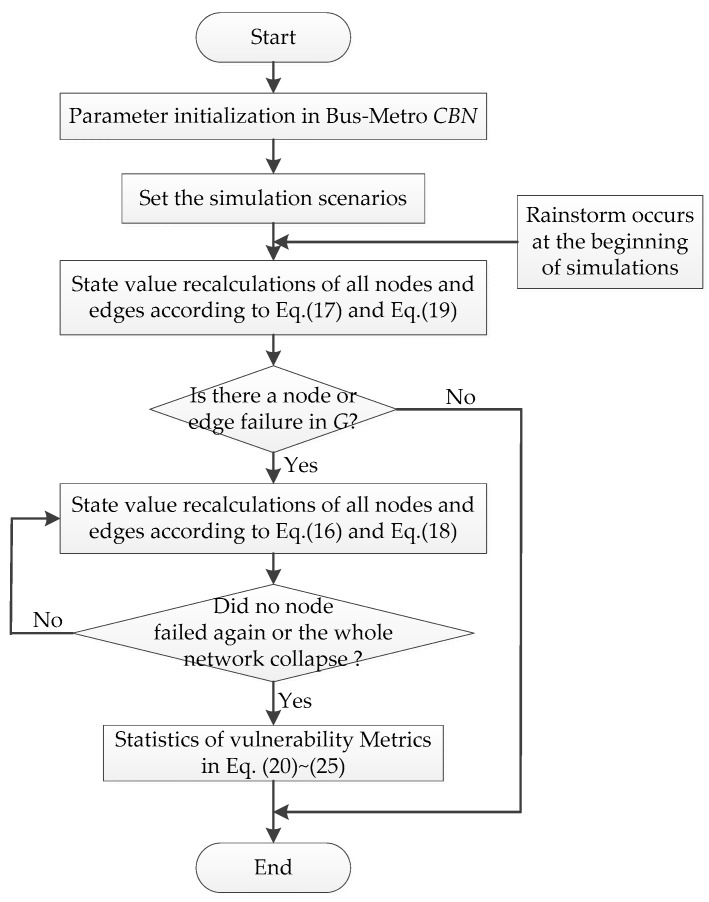
The simulation process.

**Figure 8:**
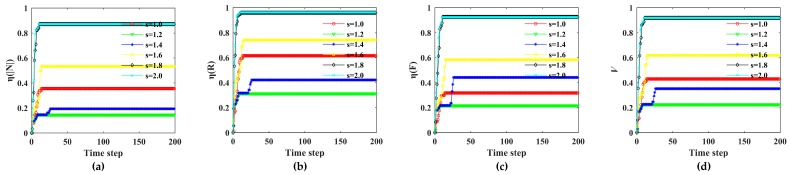
Cascading failures of Bus–Metro CBN under different rainstorm intensities. (**a**)–(**c**) show the loss ratio of node scale in the maximum connected subgraph, the network efficiency and the passenger flow with time under different rainstorm intensities, respectively. (**d**) shows the change of network vulnerability with time under different rainstorm intensities. Note: In these scenarios, *α* = 0.3, *ε*_1_ = 0.2, *ε*_2_ = 0.2, *λ*_1_ = *λ*_2_ = *λ*_3_ = $\frac{1}{3}$.

**Figure 9:**
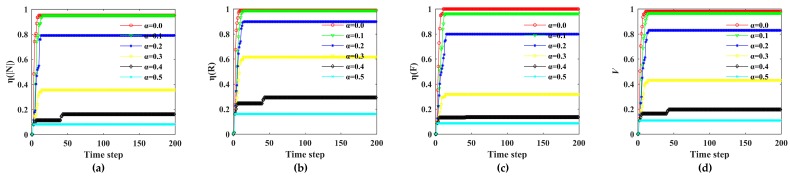
Cascading failures of Bus–Metro CBN under different capacity tolerances. (**a**)–(**c**) show the loss ratio of node scale in the maximum connected subgraph, the network efficiency and the passenger flow with time under different capacity tolerances, respectively. (**d**) shows the change of network vulnerability with time under different capacity tolerances. Note: In these scenarios, *s* = 1.0, *ε*_1_ = 0.2, *ε*_2_ = 0.2, *λ*_1_ = *λ*_2_ = *λ*_3_ = $\frac{1}{3}$.

**Figure 10:**
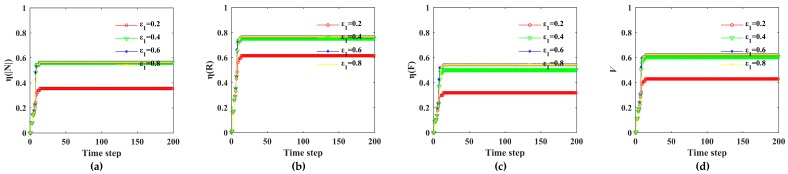
Cascading failures of Bus–Metro CBN under different node coupling strengths. (**a**)–(**c**) show the loss ratio of node scale in the maximum connected subgraph, the network efficiency and the passenger flow with time under different node coupling strengths, respectively. (**d**) shows the change of network vulnerability with time under different node coupling strengths. Note: in these scenarios, *s* = 1.0, *α* = 0.3, *ε*_2_ = 0.2, $\lambda_{1}=\lambda_{2}=\lambda_{3}=\frac{1}{3}$.

**Figure 11:**
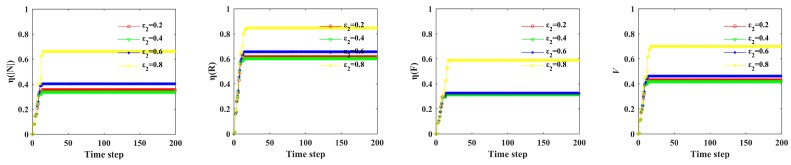
Cascading failures of Bus–Metro CBN under different edge coupling strengths. (**a**)–(**c**) show the loss ratio of node scale in the maximum connected subgraph, the network efficiency and the passenger flow with time under different edge coupling strengths, respectively. (**d**) shows the change of network vulnerability with time under different edge coupling strengths. Note: in these scenarios, *s* = 1.0, *α* = 0.3, *ε*_1_ = 0.2, $\lambda_{1}=\lambda_{2}=\lambda_{3}=\frac{1}{3}$.

The influence of a rainstorm on the Bus–Metro CBN can be divided into direct and indirect effects. On one hand, the rainstorm directly affected the ground bus network. On the other hand, the transfer passengers caused by rainstorm indirectly affects the metro network. We set the initial state values of all nodes and edges to (0,1) and regard a rainstorm as an external disturbance at the beginning of simulations. Then, in line with the scenarios in Table 4, the cascading failure processes of the Bus–Metro CBN in the area encircled by Xi’an City wall were simulated according to the Equations (16)–(19). Next, the impacts of rainstorm intensity, capacity tolerance, node coupling strength and edge coupling strength on the Bus–Metro CBN were analyzed using Equations (20)–(25), this process is shown in Figure 7. The simulation results are shown in Figure 8, Figure 9, Figure 10 and Figure 11. At the same time, we have counted the convergence time (i.e., the duration when there are no nodes or edges failure) of cascading failure process under different conditions, and recorded the values of *η*(|*N*|), *η*(*R*), *η*(*F*) and V at the convergence time, which are shown in Table 5. The scenarios in Table 5 correspond to that in Table 4.

As shown in Figure 8, Figure 9, Figure 10 and Figure 11, most of the curves have only one rising process, but some curves have two rising processes, such as *s =* 1.4 in Figure 8 and *α* = 0.4 in Figure 9. This phenomenon means that when *s =* 1.4 or *α* = 0.4, the Bus–Metro CBN will have two cascading failure processes, which increases the network vulnerability. This is quite different to previous results on the cascading failures of public transport networks [59]. The two-stage cascading failure process is because that the capacity of the node or edge directly linked to the failure node or edges is large, while the capacity of the distant node or edge is small. Therefore, when the passenger flow of failed node or edge can be transferred to the distant node and edge, the second-stage cascading failure processes occurs after a period of propagation. The emergence of the two-stage cascading failure phenomenon, on one hand, points out that the Bus–Metro CBN may experience two failure process during a rainstorm, with a significant adverse effect on the operation of the Bus–Metro CBN. On the other hand, it is possible to carry out emergency repair at failed bus stops (or metro stations) and lines in between two failure processes in order to prevent new failures. 

Through comparing *η*(|*N*|), *η*(*R*), *η*(*F*), and *V* under different rainstorm circumstances (Figure 8), we found that in most scenarios, the failure consequences of the Bus–Metro CBN increase with the increase of *s*. But when *s* increases from 1.0 to 1.2, the failure consequences decrease. This is mainly because when the rainstorm intensity increases, some key nodes or edges of passenger flow transfer are more likely to fail, resulting in the inability of the passenger flow to transfer to the distant nodes or edges, thereby narrowing the influence range of node and edge failures. In addition, when *s* = 1.8 and *s* = 2.0, the cascading failure consequences of Bus–Metro CBN are almost the same, and the entire network almost collapses, which means *s* = 1.8 is the rainstorm intensity threshold that causes the Bus–Metro CBN to collapse.

From Figure 9, we find that as the tolerance parameter *α* increases, the failure consequences of Bus–Metro CBN are declining. This is because when the capacity increases, the failure probability of nodes and edges due to passenger overload will decrease, and the consequences of cascading failure will also decrease. When *α* = 0.0 and *α* = 0.1, the failure proportion is almost 100%, and the whole network almost collapses, which indicates that the Bus–Metro CBN is extremely vulnerable when the capacity of nodes and edges is equal to or close to their initial load.

As shown in Figure 10, when *ε*_1_ = 0.2, the failure consequence of Bus–Metro CBN is the smallest. When *ε*_1_ = 0.4, *ε*_1_ = 0.6 and *ε*_1_ = 0.8, the consequences of cascading failure are almost the same, and they are both larger than that in *ε*_1_ = 0.2, which means *ε*_1_ = 0.4 is the threshold of node coupling strength. When *ε*_1_ ≥ 0.4, the cascading failure consequences of Bus–Metro CBN hardly change with the increase of *ε*_1_.

Considering the cascading failures of Bus–Metro CBN under different edge coupling strength (Figure 11), we can find that when *ε*_2_ ≤ 0.6, the failure consequences of Bus–Metro CBN are almost the same in different scenarios. When *ε*_2_ = 0.8, the failure consequences of cascading failure are the largest. This shows that the threshold of edge coupling strength is *ε*_2_ = 0.6, and on the other hand, the edge coupling strength has a limit in reducing the vulnerability of Bus–Metro CBN, i.e., the vulnerability of Bus–Metro CBN will not be lower than that in *ε*_2_ = 0.6.

**Figure 12:**
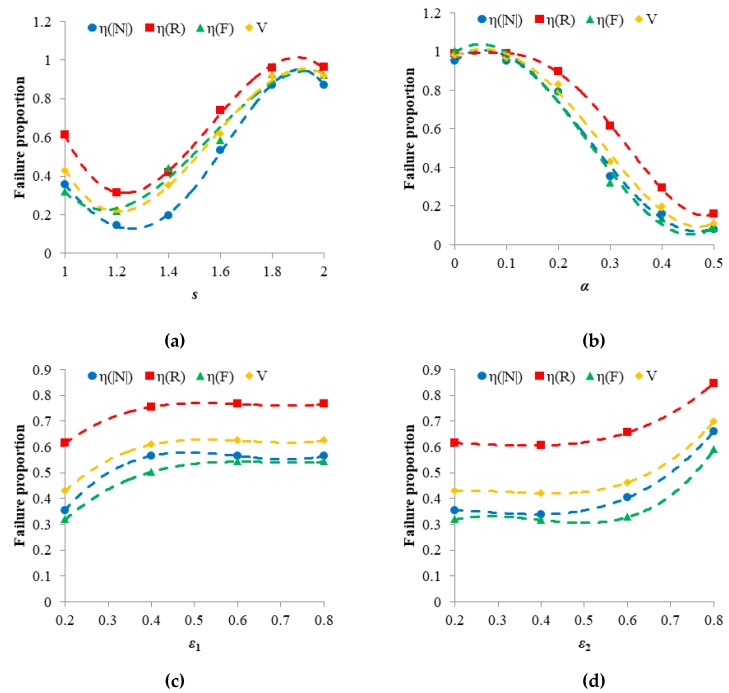
The change of *η*(|*N*|), *η*(*R*), *η*(*F*) and *V* changes with *s*, *α*, *ε*_1_, and *ε*_2_. (**a**) *α* = 0.3, *ε*_1_ = *ε*_2_ = 0.2; (**b**) *s =* 1.0, *ε*_1_ = *ε*_2_ = 0.2; (**c**) *s =* 1.0, *α =* 0.3, *ε*_2_ = 0.2; (**d**) *s =* 1.0, *α =* 0.3, *ε*_1_ = 0.2. Data is derived from Figure 8, Figure 9, Figure 10 and Figure 11 and Table 5.

To further explore the effects of rainstorm intensity, capacity tolerance, node coupling strength and edge coupling strength on the Bus–Metro CBN, we drew Figure 12 based on Figure 8, Figure 9, Figure 10 and Figure 11 and Table 5.

Figure 12a shows the relationships between *η*(|*N*|), *η*(*R*), *η*(*F*), *V* and *s*. The figure exhibits a trend of “descending-ascending-unchanged”. This means that the vulnerability of Bus–Metro CBN is fluctuating with increasing rainstorm intensity, and there is a rainstorm intensity threshold (*s* = 1.8) that cause the whole network to collapse. From observing the slope of these curves when *s* = 1.6, the slope of these curves is the largest, which means that the cascading failure consequences of Bus–Metro CBN increase the fastest in s = 1.6. Therefore, when the rainstorm intensity *s* exceeds 1.4, the traffic management department should quickly escalate emergency management and implement rescue measures to prevent the collapse of the Bus–Metro CBN.

From Figure 12(b), it is observed that *η*(|*N*|), *η*(*R*), *η*(*F*), *V* decrease with *α*, meaning that the increase in node and edge capacity can reduce the vulnerability of Bus–Metro CBN. At the same time, the decline rate of the failure proportion first increases and then decreases, which indicates that when *α* is small (*α* = 0.0) or large (*α* = 0.4), the increase in node and edge capacity has less effect on the mitigation of Bus–Metro CBN vulnerability. In addition, when *α* = 0.3, the curves have the fastest falling speed, which denote that when the node and edge capacity is 1.3 times of their initial load, increasing the node and edge capacity have the greatest effect on reducing the network vulnerability. If we consider the cost of increasing node and edge capacity, *α* = 0.3 is the point with the highest marginal benefit. Comprehensively considering the network vulnerability reduction and its cost, *α* = 0.4 is the best choice that is when the node and edge capacity is 1.4 times of their initial load.

Considering the relationships between *η*(|*N*|), *η*(*R*), *η*(*F*), *V* and *ε*_1_ (Figure 12c), it shows that the impact of *ε*_1_ on the failure proportion can be divided into two intervals, *ε*_1_ < 0.4 and *ε*_1_ ≥ 0.4, respectively. When *ε*_1_ < 0.4, the failure proportion increases as *ε*_1_ increases. When *ε*_1_ ≥ 0.4, the failure proportion is stable, and the changes of *η*(|*N*|), *η*(*R*), *η*(*F*), *V* do not exceed 5%. On the one hand, the Bus–Metro CBN should have a low node coupling strength to reduce its vulnerability, on the other hand, only when the node coupling strength is below 0.4, the reduction of node coupling strength reduces Bus–Metro CBN vulnerability.

In terms of the relationships between *η*(|*N*|), *η*(*R*), *η*(*F*), *V* and *ε*_2_ (Figure 12d), we can also divide the effect of *ε*_2_ on the cascading failure process, *ε*_2_ ≤ 0.6 and *ε*_2_ > 0.6, respectively. When *ε*_2_ ≤ 0.6, the cascading failure processes are almost the same, and the failure proportion is stable. When *ε*_2_ > 0.6, the failure proportion increases as *ε*_2_ increases. This phenomenon indicates that *ε*_2_ has a limit to the reduction of Bus–Metro CBN vulnerability, i.e., when other conditions remain unchanged and only *ε*_2_ is allowed to be adjusted, the vulnerability *V* of Bus–Metro CBN can only be reduced to about 0.4. From the comparison of *ε*_1_ and *ε*_2_, the reduction of node coupling strength has more potential to reduce the Bus–Metro CBN vulnerability than that of edge coupling strength. Therefore, more attention should be paid to the reduction of node coupling strength.

**Figure 13:**
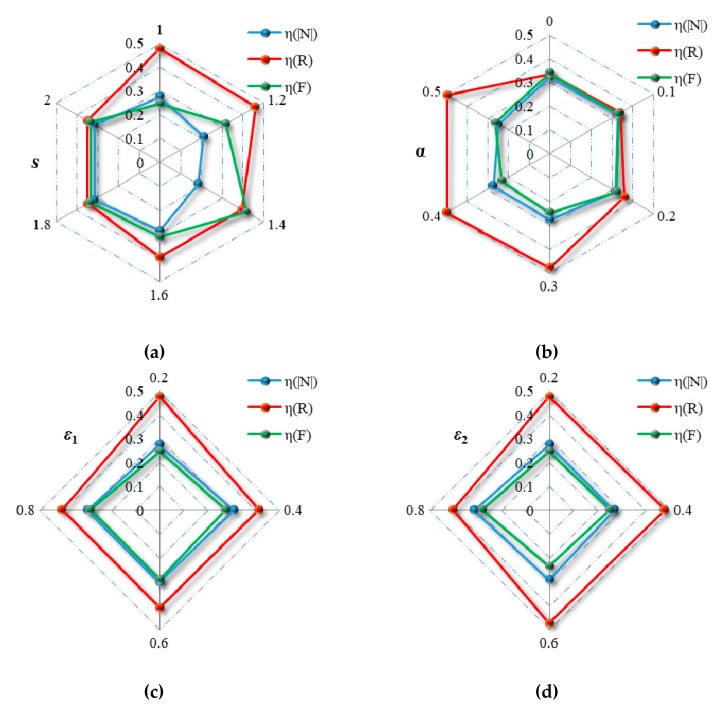
Contribution rate of *η*(|*N*|), *η*(*R*) and *η*(*F*) to *V* in different conditions. (**a**) *α* = 0.3, *ε*_1_ = *ε*_2_ = 0.2; (**b**) *s =* 1.0, *ε*_1_ = *ε*_2_ = 0.2; (c) *s =* 1.0, *α* = 0.3, *ε*_2_ = 0.2; (**d**) *s =* 1.0, *α* = 0.3, *ε*_1_ = 0.2. Data is derived from Figure 8, Figure 9, Figure 10 and Figure 11 and Table 5.

In addition, to analyze the main factor affecting the vulnerability of the Bus–Metro CBN, we calculated the contribution rates (i.e., the influence of different indicators on the formation of results) of *η*(|*N*|), *η*(*R*) and *η*(*F*) to *V* based on the data in Figure 8, Figure 9, Figure 10 and Figure 11 and Table 5, and the results are shown in Figure 13. Figure 13 shows that in most of the scenarios, the contribution rate of *η*(*R*) to *V* was higher than that of *η*(|*N*|) and *η*(*F*) to *V*. This means that the vulnerability of the Bus–Metro CBN under a rainstorm was affected mainly by whole-network efficiency, i.e., the performance of network connectivity.

As shown in Figure 13a, when *s* < 1.4, the vulnerability of Bus–Metro CBN is mainly caused by the decline of *η*(*R*); when *s* ≥ 1.8, the contribution of *η*(|*N*|), *η*(*R*), *η*(*F*) to *V* are almost equal. However, when *s* = 1.4, *η*(*F*) has the biggest impact on the vulnerability of Bus–Metro CBN. This means that the vulnerability of the Bus–Metro CBN was mainly caused by the destruction of the topological structure under less severe rainstorm. When the rainstorm was more severe, the failure of the passenger flow also has a great influence on the vulnerability of the Bus–Metro CBN. The main reason for this is that passengers at failed bus stops (or metro stations) and lines may transfer to other normally functioning bus stops (or metro stations) and lines, thus reducing passenger loss in the system as a whole. However, given that the topology of the system is almost static and lacks a dynamic self-healing ability, i.e., the topology cannot repair itself, this will greatly affect the system’s vulnerability.

Under the conditions of different *α* (Figure 13b), the contributions of *η*(|*N*|) and *η*(*F*) to *V* are almost the same. At the same time, when *α* ≤ 0.2, the contribution of *η*(*R*) to *V* is almost equal to that of *η*(|*N*|) and *η*(*F*) to *V.* But when *α* > 0.2, the contribution of *η*(*R*) to *V* far exceeds that of *η*(|*N*|) and *η*(*F*) to *V.* These results indicate that when the node and edge capacity is relatively small (*α* ≤ 0.2), the topology structure and passenger flow are almost the same important to the Bus–Metro CBN. But when the node and edge capacity is relatively large (*α* > 0.2), the destruction of the topological structure is the main cause of vulnerability in the Bus–Metro CBN, so the topological structure should be given more protection in these scenarios.

Figure 13c shows that under different *ε*_1_, the impact of *η*(|*N*|) and *η*(*F*) on *V* are almost the same. At the same time, it is obvious that *η*(*R*) is always the main factor that affects *V*. Figure 13d shows results similar to Figure 13c. These results imply that when other conditions are given, the degradation of topology structure is always the main cause of Bus–Metro CBN vulnerability, regardless of the degree of connection between different nodes and different edges.

Many previous topology-based researches of network robustness or vulnerability found that the topology is important to the network [97,98,99]. When the topology of the network degrades due to node or edge failures, the performance of the entire network is greatly reduced. In this paper, the loads in nodes and edges are passengers which means the proposed CMLs model is a flow-based model [33,34]. Through this model, we can compare the impacts of topology structure integraty and passenger flow on the network vulnerability in light of the topology-based metrics of *η*(|*N*|), *η*(*R*) and the flow-based metric of *η*(*F*). According to the analysis of the main factors affecting the vulnerability in Bus–Metro CBN, although the topology is still important to the network in the flow-based cascading failure model, in some scenarios (*s* = 1.4, *s* ≥ 1.8, *α* ≤ 0.2), the passenger flow will also play a pivotal role in the network performance.

## 5. Conclusions and Recommendations

Due to the high incidence of rainstorms and their great impact on urban public traffic, this study empirically examined the impact of rainstorms on the Bus–Metro CBN that consists of ground bus and metro using vulnerability calculations. Firstly, we established a way of transforming the rainstorm intensity into the quantitative impact on the Bus–Metro CBN. Then based on the CML model in the research of Huang, Peng, and Ren [64,65,66], we proposed a flow-based CML model to simulate the cascading failures in the Bus–Metro CBN. At the same time, we considered not only the state failure of nodes and edges, but also the failure caused by flow overload of nodes and edges. Through the topology-based and flow-based metrics, a vulnerability operator is built to quantitatively calculate the vulnerability of Bus–Metro CBN. Finally, these models and methods are used in an empirical case and results were obtained.

In summary, through the empirical study, we found that the Bus–Metro CBN may experience a secondary failure process due to the capacity of nodes and edges. This indicates that the network will have two-stage failure processes at different times and thus cause the consequences of the cascading failure to worsen over time. However, from the perspective of emergency management, the multi-stage failure process provides time for the repair of failed bus stops (or metro station) and lines. Managers can take repair measures between two failure processes, and thus prevent a new round of failure. 

Considering the impact of rainstorm intensity on the vulnerability of Bus–Metro CBN, we found that the consequence of cascading failure does not always increase as the rainstorm intensity increases. The relationship between failure proportion and rainstorm intensity shows a trend of “descending–ascending–unchanged”, which means on the one hand the traffic management department should take more management and rescue measures during the “ascending” period. On the other hand, there is a rainstorm intensity threshold that causes the Bus–Metro CBN to collapse, which can be used as a warning for rainstorm observations. 

For reducing the vulnerability of Bus–Metro CBN, we studied the impact of capacity increases on the network vulnerability, and found that the decline rate in network vulnerability increases first and then decreases as the capacity increases. At the same time, with simultaneous consideration for the capacity increase and its cost, we obtained the optimal capacity of nodes and edges, which can be used to support public transport planning.

In terms of the coupling strength of Bus–Metro CBN, the network vulnerability increases as the node coupling strength and edge coupling strength increase. However, the influence of node coupling strength on network vulnerability has an upper limit, and the edge coupling strength has a lower limit on network vulnerability. In contrast, node coupling strength has greater potential to reduce the vulnerability of Bus–Metro CBN than edge coupling strength. Therefore, in the actual Bus–Metro CBN, the coupling relationship between bus stops (metro stations) should be prioritized to minimize them.

Moreover, from the contribution to the vulnerability of Bus–Metro CBN, the decline of the whole network efficiency contributes the largest to the network vulnerability in most scenarios. But in some scenarios, such as when the capacities of nodes and edges are small, the decline of passenger flow also contributes greatly to the vulnerability of Bus–Metro CBN. In general, the traffic management department should give priority to ensuring the connectivity of the shortest path between the stop (station) pairs in the Bus–Metro CBN to reduce its vulnerability.

In this study, the urban ground bus and metro systems were abstracted into a complex bilayer network, and both the failure of nodes and edges in the network were considered. The vulnerability of the Bus–Metro CBN under rainstorm was reflected by the node scale of the maximum connected subgraph, the whole-network efficiency and the passenger volume. Based on the CMLs model, the cascading failures of the Bus–Metro CBN were simulated, and some noteworthy results were different from those yielded by previous studies. These results enabled us to provide the aforementioned recommendations for reducing the vulnerability of the Bus–Metro CBN. In future research, we recommend further exploration of strategies for the repair of cascading failures in a Bus–Metro CBN under different conditions, and the relationship between the contact enhancements of the network’s topological structure and the degree of failure of the network function.

## Figures and Tables

**Table 1:** The influence of different intensities of rainfall on Bus–Metro CBN [57].

Intensities of Rainfall	Influence on Bus–Metro CBN Operation
≥50 mm/24 h	Reduces the friction coefficient of road surfaces and increases the incidence of bus accidents.
≥100 mm/24 h	Reduces the friction coefficient of road surfaces and increases the incidence of bus accidents; partial rail transit interruptions and delay.
≥150 mm/24 h	Bus outage, delays, bus lines shrinkage; partial rail transit interruptions and delay, with orbiting system destroyed.

**Table 2:** Key data on the Bus–Metro CBN.

Bus Stop	Bus Lines Operating at the Stop	Whether is a Transfer Stop
BAISHULIN	258, 309, 706, 707	No
HONGHUJIE	102, 103, 10, 12, 235, 28, 301, 506, 606, 702	Yes
…	…	…
LUOMASHI	706, 707	No
XIWUYUAN	107, 703, 702	No
Bus line	No. of bus stops on the line	No. of transfer stops on the line
4	8	3
20	4	1
…	…	…
182	8	1
606	8	5
Metro station	Metro lines running through station	Whether is a transfer station
ANYUANMEN	2	No
BEIDAJIE	1, 2	Yes
…	…	…
ZHONGLOU	2	No
WULUKOU	1	No
Metro line	No. of metro stations on the line	No. of transfer stations on the line
1	5	1
2	4	1

Note: All data pertain only to the area encircled by Xi’an City wall.

**Table 3:** Feature parameters of Xi’an Bus–Metro CBN and corresponding E-R random network.

Network	Nodes	Edges	Average Degree	Average Path Length	Clustering Coefficient
Bus	54	85	3.148	4.593	0.201
Metro	8	7	1.750	2.286	0.000
Bus–Metro	62	99	3.194	4.480	0.174

**Table 4:** Simulation scenarios.

Scenario	*s*	*α*	*ε*_1_	*ε*_2_	Object of Research
1	1.00	0.3	0.2	0.2	Impact of rainstorm on Bus–Metro CBN
2	1.20	0.3	0.2	0.2	
3	1.40	0.3	0.2	0.2	
4	1.60	0.3	0.2	0.2	
5	1.80	0.3	0.2	0.2	
6	2.00	0.3	0.2	0.2	
7	1.00	0.0	0.2	0.2	Impact of capacity tolerance on Bus–Metro CBN
8	1.00	0.1	0.2	0.2	
9	1.00	0.2	0.2	0.2	
10	1.00	0.3	0.2	0.2	
11	1.00	0.4	0.2	0.2	
12	1.00	0.5	0.2	0.2	
13	1.00	0.3	0.2	0.2	Influence of node coupling strength on Bus–Metro CBN
14	1.00	0.3	0.4	0.2	
15	1.00	0.3	0.6	0.2	
16	1.00	0.3	0.8	0.2	
17	1.00	0.3	0.2	0.2	Influence of edge coupling strength on Bus–Metro CBN
18	1.00	0.3	0.2	0.4	
19	1.00	0.3	0.2	0.6	
20	1.00	0.3	0.2	0.8	

**Table 5:** Changes in feature indices of cascading failure in the Bus–Metro CBN.

Scenario	Variable	Value of Variable	Convergence Time (Step)	*η*(|*N*|)	*η*(*R*)	*η*(*F*)	*V*
1	*s*	1.00	14	0.355	0.615	0.318	0.429
2		1.20	8	0.145	0.314	0.217	0.227
3		1.40	26	0.194	0.421	0.441	0.352
4		1.60	15	0.532	0.740	0.583	0.618
5		1.80	11	0.871	0.959	0.926	0.919
6		2.00	10	0.871	0.962	0.923	0.919
7	*α*	0.0	11	0.952	0.989	1.000	0.980
8		0.1	15	0.952	0.988	0.964	0.968
9		0.2	15	0.790	0.898	0.799	0.829
10		0.3	14	0.355	0.615	0.318	0.429
11		0.4	43	0.161	0.293	0.136	0.197
12		0.5	2	0.081	0.161	0.086	0.109
7	*ε*_1_	0.2	14	0.355	0.615	0.318	0.429
8		0.4	14	0.565	0.755	0.503	0.608
9		0.6	14	0.565	0.766	0.542	0.624
10		0.8	14	0.565	0.766	0.542	0.624
11	*ε*_2_	0.2	14	0.355	0.615	0.318	0.429
12		0.4	14	0.339	0.605	0.316	0.420
13		0.6	14	0.403	0.656	0.327	0.462
14		0.8	19	0.661	0.847	0.589	0.699
